# TyG indicator–TyHGB and the risk of gestational diabetes mellitus: a retrospective cohort study

**DOI:** 10.3389/fnut.2026.1967930

**Published:** 2026-10-19

**Authors:** Xiaohua Li, Weizhen Bu, Wenjing Liang, Ziling Ji, Yunfei Chen, Yan Xuan, Xiaojing Hu

**Affiliations:** 1 Department of Nursing, Hainan Women and Children's Medical Centre, Haikou, China; 2 Department of Paediatric Urology, Hainan Women and Children's Medical Centre, Haikou, China; 3 Department of Nursing, Children’s Hospital of Fudan University, National Children's Medical Center, Shanghai, China

**Keywords:** cohort study, gestational diabetes, glucose, pregnancy, triglycerides

## Abstract

**Objective:**

The objective was to explore the association between triglyceride-high-density lipoprotein cholesterol-glucose body index (TyHGB) and gestational diabetes mellitus (GDM) among Chinese pregnant women.

**Methods:**

This retrospective cohort study enrolled 1,063 pregnant women who underwent GDM screening using the oral glucose tolerance test (OGTT). The TyHGB was calculated according to the following formula: TyHGB = TG/HDL-C + 0.7*fasting plasma glucose (FPG) (mmol/L) + 0.1*pre-pregnancy body mass index (pre-BMI, kg/m^2^). Multivariate logistic regression was performed to calculate odds ratios (ORs) and 95% confidence intervals (CIs) and evaluate the association between TyHGB and GDM among Chinese pregnant women. We performed subgroup analyses as well as receiver operating characteristic (ROC) curve analysis to evaluate the diagnostic efficacy of TyHGB for GDM.

**Results:**

Of the total study participants, 190 women (17.87%) were newly diagnosed with GDM. The median age of the cohort was 31.00 years (IQR, 28.0–34.0). After adjusting for relevant confounding factors, multivariable logistic regression revealed that increased TyHGB levels was significantly associated with increased GDM risk, with an odds ratio (OR) of 1.82 (95% CI: 1.37–2.44). In addition, the area under the receiver operating characteristic curve (AUC) for TyHGB was 0.675 (95% CI: 0.646–0.703). The optimal cut-off value was 5.995, yielding a specificity of 63.12% and a sensitivity of 66.32%. Notably, the TyHGB exhibited superior diagnostic performance for GDM relative to maternal age, pre-BMI, FPG and the triglyceride-glucose index.

**Conclusion:**

The TyHGB represents a promising indicator for the identification of pregnant women at high risk of GDM.

## Introduction

Gestational diabetes mellitus (GDM), one of the most common metabolic complications during pregnancy, defined as newly onset glucose intolerance in gestation (1, 2). GDM substantially increases the risks of adverse maternal and fetal outcomes and contributes to both short-term and long-term metabolic disorders in both mothers and infants (3–6). The global prevalence of GDM has soared in recent decades, impacting roughly 15% of pregnancies worldwide (7). The overall prevalence in China is 14.8% (8), with marked regional variations across cities. Relevant studies conducted among pregnant women in various regions of China have reported GDM prevalence rates of 15.2% (9), 19.7% in Beijing (10), and 10.5% in Changsha (11). Thus, early detection and timely intervention of GDM are critical to reducing pregnancy-related complications and improving maternal and neonatal outcomes.

Currently, the oral glucose tolerance test (OGTT) performed at 24–28 weeks of gestation remains the standard diagnostic approach for GDM (12). However, this late-gestation screening strategy has notable limitations. Delayed diagnosis fails to enable early preventive interventions, potentially leading to irreversible adverse pregnancy outcomes (13–15). In addition, routine OGTT screening imposes considerable time and economic burdens on pregnant populations (16–18). Therefore, it is urgent to establish simple and reliable early biomarkers for GDM risk stratification and early intervention.

The triglyceride-high-density lipoprotein cholesterol-glucose body (TyHGB) index is a novel and comprehensive metabolic indicator that integrates triglyceride (TG), high-density lipoprotein cholesterol (HDL-C), fasting plasma glucose (FPG), and pre-pregnancy body mass index (pre-BMI) (19, 20). Previous studies have confirmed that TyHGB performs well in predicting insulin resistance (20). GDM is pathologically similar to type 2 diabetes mellitus, both mainly caused by pancreatic β-cell dysfunction and increased insulin resistance (21–23). Based on the common metabolic mechanism, the relationship between TyHGB and GDM has attracted increasing attention. Although a previous study has verified the predictive value of TyHGB for GDM in Chinese pregnant women, it did not adjust for the metabolic effects of assisted reproductive technology (24). Accordingly, this study aimed to evaluate the predictive value of TyHGB for GDM and explore its feasibility as an ideal alternative biomarker for early screening of GDM.

## Materials and methods

### Study population

In this retrospective cohort study, pregnant women delivering between January 2024 and December 2024 who had undergone first-trimester lipid profile testing were included. Subjects with gestational age below 14 weeks were retrieved from electronic medical records, and their pregnancy outcomes were collected up to the time of delivery. After strict exclusion, a total of 1,063 singleton pregnant women were ultimately included in the final analysis. To minimize selection bias, we excluded women with multiple pregnancies, incomplete medical records, endocrine disorders, severe systemic diseases, pre-pregnancy type 1/type 2 diabetes mellitus, long-term use of drugs interfering with glucose metabolism, chronic hypertension and other pregnancy complications, as shown in Figure 1.

**Figure 1:**
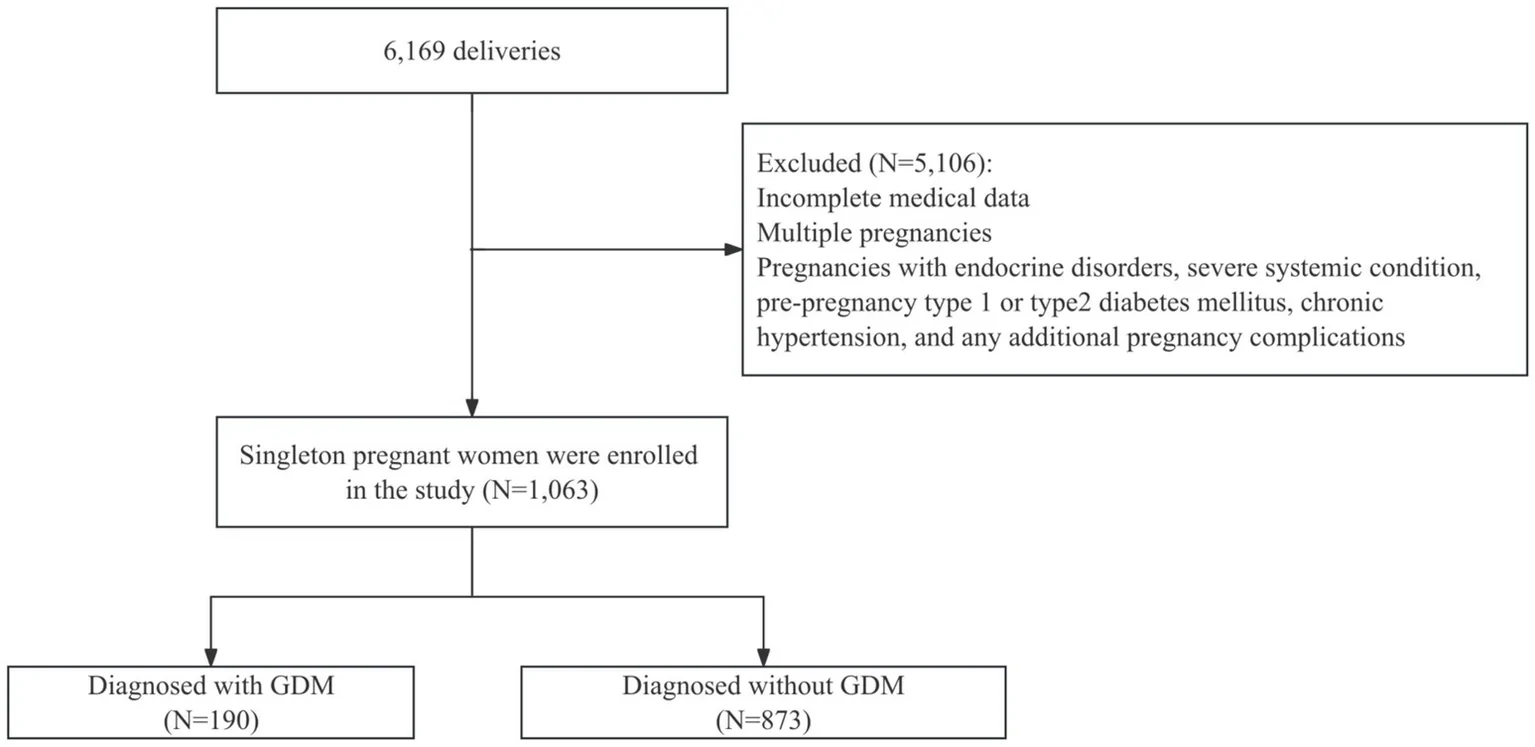
Flowchart of participant screening and grouping. A total of 6,169 delivery records were initially screened. A total of 5,106 cases were excluded according to predefined exclusion criteria, and 1,063 singleton pregnant women were finally enrolled. The enrolled subjects were divided into the GDM group (*N* = 190) and non-GDM group (*N* = 873) based on the diagnostic results of gestational diabetes mellitus.

### Measurements and definitions

#### Study variables definition

All key study variables were clearly defined according to standardized methodological specifications. The primary exposure variable was the first-trimester TyHGB level. The primary outcome variable was GDM diagnosed at 24–28 weeks of gestation. Predictors and potential confounders included maternal age, pre-pregnancy BMI, assisted reproductive conception, gravidity, history of abortion, ALT, AST, total TC, TG, LDL-C, HDL-C, FPG, and HbA1c. These covariates were selected based on clinical plausibility and previous literature regarding GDM risk factors.

#### Data collection

Trained researchers systematically collected complete clinical data of all participants based on institutional electronic medical records. The selection of covariates was guided by clinical experience and a comprehensive review of previous studies focusing on GDM risk factors. Collected variables included ethnicity, maternal age, pre-BMI, assisted reproductive conception, history of abortion, gravidity, and parity. We also incorporated additional established confounding factors that may influence the study outcome. Fasting venous blood samples were obtained after an overnight fast. Laboratory indicators tested encompassed alanine aminotransferase (ALT), aspartate aminotransferase (AST), TC, TG, LDL-C, HDL-C, FPG, and glycated hemoglobin (HbA1c). Pre-BMI was calculated as pre-pregnancy weight (kg) divided by height squared (m^2^).

#### Measurement of TyHGB and TyG

TyHGB = (TG/HDL-C) + 0.7 × FPG + 0.1 × BMI (24). TyG = ln([TG × FPG]/2) (24).

#### Diagnosis of GDM

All participants underwent the 75 g OGTT for GDM screening at 24–28 weeks of gestation. In accordance with the International Association of the Diabetes in Pregnancy Study Groups (IADPSG) criteria, GDM was diagnosed when any of the following cut-off values were satisfied: fasting plasma glucose (0-h OGTT) ≥ 92 mg/dL, 1-h post-load glucose ≥180 mg/dL, or 2-h post-load glucose ≥153 mg/dL (25).

### Statistical analyses

Continuous variables were expressed as median (interquartile range, IQR), and between-group comparisons were performed using the Kruskal–Wallis test. Categorical variables were presented as numbers (percentages) and compared via the Pearson’s chi-squared test (Figure 1).

Binary logistic regression models were applied to evaluate the association between TyHGB and GDM risk with four incremental adjustment levels. The crude model included no covariates. Model 1 was adjusted for maternal age, assisted reproductive conception, and gravidity. Model 2 was additionally adjusted for TC, LDL-C, ALT, AST and HbA1c; Model 3 incorporated all aforementioned covariates for fully adjusted analysis. TyHGB was categorized into tertiles according to its empirical distribution within the study population; no external published cut-off values were used. After categorizing TyHGB into tertiles, a linear trend test was performed across tertile groups. Subgroup analyses were conducted using unadjusted models to prevent over adjustment bias. Receiver operating characteristic (ROC) analysis was utilized to calculate the sensitivity, specificity, and optimal cut-off value of TyHGB for GDM prediction. Subsequently, a linear trend assessment was performed on the model after evaluating the TyHGB by tertiles. The diagnostic discriminatory ability was quantified using the area under the receiver operating characteristic curve (AUC) with bootstrap validation. The DeLong test was adopted to compare differences in AUC values among different indicators.

All statistical analyses were conducted using R software (version 4.2.0, R Foundation for Statistical Computing, Vienna, Austria) and EmpowerStats software (version 4.2). A two-sided *p* value < 0.05 was defined as the threshold for statistical significance.

This retrospective study was approved by the Ethics Committee of Hainan Women and Children’s Medical Centre (Ethics approval number: HNWCMC2026159). The requirement for individual informed consent was waived by the ethics committee, since all clinical data were fully anonymized and identifying information was removed.

## Results

### Baseline characteristics

A total of 1,063 pregnant women were enrolled in this cohort, of whom 190 (17.87%) were diagnosed with GDM. Most participants were of Han ethnicity (96.05%), and 49.67% were primigravida. Baseline characteristics of all participants are presented in Table 1.

**Table 1:** Characteristics of participants.

**Characteristics**	**Overall**	TyHGB			Statistic	*p* value
		T1 (4.40, 5.59)	T2 (5.60, 6.16)	T3 (6.17, 10.71)		
Participants	1,063	349	358	356		
Age (years)	31.00 (28.00, 34.00)	30.00 (27.00, 33.00)	31.00 (28.00, 34.00)	31.00 (29.00, 34.00)	30.18	<0.001
Pre-pregnancy BMI (kg/m^2^)	26.17 (24.34, 28.40)	24.84 (23.01, 26.17)	26.48 (24.69, 28.40)	27.94 (25.78, 30.14)	210.10	<0.001
TC (mmol/L)	179.82 (160.48, 199.15)	173.24 (156.61, 193.35)	179.82 (160.58, 198.66)	183.30 (163.96, 204.76)	15.28	<0.001
TG (mmol/L)	98.24 (73.46, 131.87)	79.65 (61.95, 99.12)	93.81 (73.46, 119.48)	138.06 (101.78, 178.11)	289.32	<0.001
HDL (mmol/L)	75.41 (64.97, 86.23)	82.37 (74.25, 94.35)	75.02 (66.13, 84.20)	67.29 (58.78, 76.95)	167.32	<0.001
LDL (mmol/L)	87.78 (73.09, 102.48)	79.27 (66.90, 92.42)	88.94 (75.02, 101.32)	95.90 (80.72, 110.21)	101.11	<0.001
ALT (mmol/L)	13.00 (10.00, 18.00)	13.00 (10.00, 16.00)	13.00 (10.00, 17.75)	14.00 (11.00, 21.00)	19.73	<0.001
AST (mmol/L)	16.00 (14.00, 18.00)	16.00 (15.00, 19.00)	16.00 (14.00, 18.00)	16.00 (14.00, 19.00)	7.59	0.023
FPG (mmol/L)	80.64 (76.05, 85.68)	75.96 (72.36, 79.38)	80.82 (77.27, 84.73)	85.86 (81.36, 93.24)	350.36	<0.001
Gestational age at delivery (weeks)	39.00 (38.00, 39.00)	39.00 (38.00, 39.00)	39.00 (38.00, 39.00)	38.00 (38.00, 39.00)	13.04	0.001
Birthweight (g)	3160.00 (2900.00,3430.00)	3100.00 (2850.00, 3350.00)	3190.00 (2935.00, 3480.00)	3220.00 (2950.00, 3462.50)	15.04	<0.001
Ethnicity						0.067
Han	1,021 (96.05%)	336 (96.28%)	344 (96.09%)	341 (95.79%)		
Li	29 (2.73%)	11 (3.15%)	12 (3.35%)	6 (1.69%)		
Others	13 (1.22%)	2 (0.57%)	2 (0.56%)	9 (2.53%)		
Assisted reproduction					6.04	0.049
No	1,038 (97.65%)	346 (99.14%)	349 (97.49%)	343 (96.35%)		
Yes	25 (2.35%)	3 (0.86%)	9 (2.51%)	13 (3.65%)		
Abortion history					5.588	0.061
No	684 (64.35%)	236 (67.62%)	236 (65.92%)	212 (59.55%)		
Yes	379 (35.65%)	113 (32.38%)	122 (34.08%)	144 (40.45%)		
Parity					51.95	<0.001
1	528 (49.67%)	218 (62.46%)	181 (50.56)	129 (36.24%)		
2	421 (39.60%)	111 (31.81%)	134 (37.43%)	176 (49.44%)		
≧3	114 (10.72%)	20 (5.73%)	43 (12.01%)	51 (14.33%)		
Gravidity					48.67	<0.001
Primiparity	528 (48.67%)	218 (62.46%)	181 (50.56%)	129 (36.24%)		
Multiparity	535 (50.33%)	131 (37.54%)	177 (49.44%)	227 (63.76%)		
Mode of delivery					4.097	0.129
Vaginal delivery	683(64.25%)	238 (68.19%)	228 (63.69%)	217 (60.96%)		
Caesarean section	380(35.75%)	111 (31.81%)	130 (36.31%)	139 (39.04%)		
GDM					55.75	<0.001
No	873 (82.13%)	326 (93.41%)	291 (81.28%)	256 (71.91%)		
Yes	190 (17.87%)	23 (6.59%)	67 (18.72%)	100 (28.09%)		

The median maternal age was 31.0 years (IQR, 28.0–34.0), and the median pre-BMI was 26.17 kg/m^2^ (IQR, 24.34–28.40). With regard to lipid profiles, the median total cholesterol (TC), TG, HDL-C, and LDL-C were 179.82 mmol/L (IQR, 160.48–199.15), 98.24 mmol/L (IQR, 73.46–131.87), 75.41 mmol/L (IQR, 64.97–86.23), and 87.78 mmol/L (IQR, 73.09–102.48), respectively.

Table 1 further stratifies baseline characteristics across TyHGB tertiles. Participants in the highest tertile (Q3) exhibited a higher prevalence of GDM, elevated maternal age, pre-BMI, TC, TG, LDL-C, ALT, AST, and FPG, alongside reduced HDL-C levels.

### Association between TyHGB and GDM

Univariate logistic regression analyses were performed to screen potential risk factors for GDM, as summarized in Table 2. Advanced maternal age, pre-BMI, TG, LDL-C, ALT, FPG, and TyHGB were positively correlated with increased GDM risk, whereas HDL-C was negatively associated with GDM prevalence. Multivariable logistic regression models were further applied to evaluate the independent association between TyHGB and GDM. The crude model demonstrated that elevated TyHGB was significantly correlated with higher GDM risk. After adjustment for maternal age, assisted reproductive conception, and gravidity in Model 1, each one-unit increment in TyHGB was associated with a 1.88-fold increased risk of GDM. Compared with participants in the lowest TyHGB quintile, those in the highest quintile exhibited substantially higher GDM susceptibility (OR = 5.22, 95% CI: 3.20–8.54). Further adjustment for TC, LDL-C, ALT, AST, and HbA1c in Model 2 did not attenuate this association, and TyHGB remained an independent risk indicator for GDM < 0.05. In the fully adjusted Model 3, TyHGB maintained a significant positive association with GDM (OR = 1.62, 95% CI: 1.29–2.03 < 0.001). Consistent across all regression models, the risk of GDM increased progressively with rising TyHGB quintiles, with a significant linear trend< 0.001 (Table 3).

**Table 2:** Results of univariate analysis.

Characteristics	Non-GDM (n = 873)	GDM (*n* = 190)	OR (95%, CI)	*p* value
Age (years)	30.00 (28.00, 33.00)	32.00 (29.00, 34.00)	1.08 (1.04, 1.12)	<0.001
Pre-pregnancy BMI (kg/m^2^)	20.83 (19.14, 22.67)	21.79 (20.09, 24.21)	1.11 (1.06, 1.17)	<0.001
TC (mmol/L)	179.43 (159.32, 199.54)	180.39 (164.73, 198.38)	1.00 (1.00, 1.01)	0.798
TG (mmol/L)	96.47 (71.69, 129.21)	109.74 (83.19, 143.82)	1.01 (1.01, 1.01)	<0.001
HDL (mmol/L)	76.18 (66.13, 87.01)	69.80 (61.20, 81.88)	0.98 (0.97, 0.99)	<0.001
LDL (mmol/L)	85.46 (71.54, 101.32)	93.38 (80.43, 105.47)	1.01 (1.01, 1.01)	0.011
ALT (mmol/L)	13.00 (10.00, 18.00)	15.00 (12.00, 21.00)	1.02 (1.01, 1.03)	0.003
AST (mmol/L)	16.00 (14.00, 18.00)	16.00 (14.00, 18.00)	1.02 (0.99, 1.04)	0.165
FPG (mmol/L)	80.10 (75.60, 84.96)	82.89 (78.30, 87.97)	1.03 (1.02, 1.04)	<0.001
Ethnicity				
Han	837 (95.88%)	184 (96.84%)	1.0	
Li	24 (2.75%)	5 (2.63%)	0.95 (0.36, 2.52)	0.914
Others	12 (1.37%)	1 (0.53%)	0.38 (0.05, 2.93)	0.352
Assisted reproduction				
No	855 (97.94%)	183 (96.32%)	1.0	
Yes	18 (2.06%)	7 (3.68%)	1.82 (0.75, 4.41)	0.187
Abortion history				
No	564 (64.60%)	120 (63.16%)	1.0	
Yes	309 (35.40%)	70 (36.84%)	1.06 (0.77, 1.47)	0.706
Parity				
1	445 (50.97%)	83 (43.68%)	1.0	
2	340 (38.95%)	81 (42.63%)	1.28 (0.91, 1.79)	0.155
≧3	88 (10.08%)	26 (13.68%)	1.58 (0.96, 2.60)	0.069
Gravidity				
Primiparity	445 (50.97%)	83 (43.68%)	1.0	
Multiparity	428 (49.03%)	107 (56.32%)	1.34 (0.98, 1.84)	0.069
TyHGB	5.79 (5.44, 6.29)	6.19 (5.83, 6.63)	1.917 (1.60, 2.30)	<0.0001

**Table 3:** Relationship between TyHGB and GDM risk in different models.

Exposure	Crude model (95%, CI, *p*)	Model 1 (95%, CI, *p*)	Model 2 (95%, CI, *p*)	Model 3 (95%, CI, *p*)
TyHGB	1.92 (1.60, 2.30) < 0.001	1.88 (1.56, 2.27) < 0.001	1.67 (1.33, 2.09) < 0.001	1.62 (1.29, 2.03) < 0.001
TyHGB tertile				
T1	1.0	1.0	1.0	1.0
T2	3.26 (1.98, 5.38) < 0.001	3.15 (1.91, 5.21) < 0.001	3.02 (1.70, 5.37) < 0.001	2.90 (1.63, 5.17) < 0.001
T3	5.54 (3.42, 8.96) < 0.001	5.22 (3.20, 8.54) < 0.001	4.36 (2.43, 7.80) < 0.001	3.97 (2.19, 7.21) < 0.001

Crude model: unadjusted.

Model 1: Adjusted for age, assisted reproduction, and gravidity.

Model 2: Adjusted for TC, LDL, ALT, AST, and HbA1c.

Model 3: Adjusted for Age, Assisted reproduction, Gravidity, TC, LDL, ALT, AST, and HbA1c.

Table 4 presents the subgroup analyses of the association between TyHGB and GDM. Overall, elevated TyHGB was significantly associated with increased odds of GDM (OR = 1.92, 95% CI: 1.60–2.30, *p* < 0.001). Stratified by age, TyHGB was positively associated with GDM both in women aged <35 years (OR = 1.93, 95% CI: 1.58–2.36, *p* < 0.001) and women ≥35 years (OR = 1.75, 95% CI: 1.13–2.70, *p* = 0.012). The interaction *p* value was 0.691, indicating no significant effect modification by maternal age. When stratified by pre-pregnancy BMI, TyHGB was significantly associated with GDM among women with pre-pregnancy BMI < 25 (OR = 2.05, 95% CI: 1.65–2.53, *p* < 0.001). In women with BMI ≥ 25, the association was attenuated and no longer statistically significant (OR = 1.16, 95% CI: 0.69–1.93, *p* = 0.575). The interaction *p* = 0.044, suggesting a statistically significant interaction between pre-pregnancy BMI and TyHGB on GDM risk. In subgroups stratified by assisted reproduction, TyHGB was significantly associated with GDM in the non-assisted reproduction group (OR = 1.91, 95% CI: 1.58–2.30, *p* < 0.001). Although the point estimate was similar in the assisted reproduction group (OR = 1.89, 95% CI: 0.67–5.33, *p* = 0.225), the result was not significant. Interaction *p* = 0.990, showing no significant interaction.

**Table 4:** Association between TyHGB and GDM in different subgroups.

Variables	*n* (%)	OR (95% CI)	*p*	*P* for interaction
All cases	1,063 (100.00)	1.92 (1.60,2.30)	<0.001	
Age				0.691
<35	940 (88.43)	1.93 (1.58, 2.36)	<0.001	
≥35	123 (11.57)	1.75 (1.13, 2.70)	0.012	
Pre-pregnancy BMI				0.044
<25	952 (89.56)	2.05 (1.65, 2.53)	<0.001	
≥25	111 (10.44)	1.16 (0.69, 1.93)	0.575	
Assisted reproduction				0.990
No	1,038 (97.65)	1.91 (1.58, 2.30)	<0.001	
Yes	25 (2.35)	1.89 (0.67, 5.33)	0.225	
Gravidity				0.010
Primiparity	528 (49.67)	2.57 (1.88, 3.51)	<0.001	
Multiparity	535 (50.33)	1.56 (1.23, 1.97)	<0.001	

OR, odds ratio; CI, confidence interval.

### ROC analysis

ROC curve analysis was performed to assess the predictive performance of TyHGB for GDM (Table 5; Figure 2). The AUC of TyHGB was 0.675 (95% CI, 0.646–0.703). Compared with other indicators including maternal age, pre-BMI, FPG and the TyG index, TyHGB yielded the largest AUC value, indicating its better diagnostic ability for GDM. The optimal cut-off value of TyHGB determined via the Youden’s index was 5.995, with a sensitivity of 66.32% and specificity of 63.12%.

**Figure 2:**
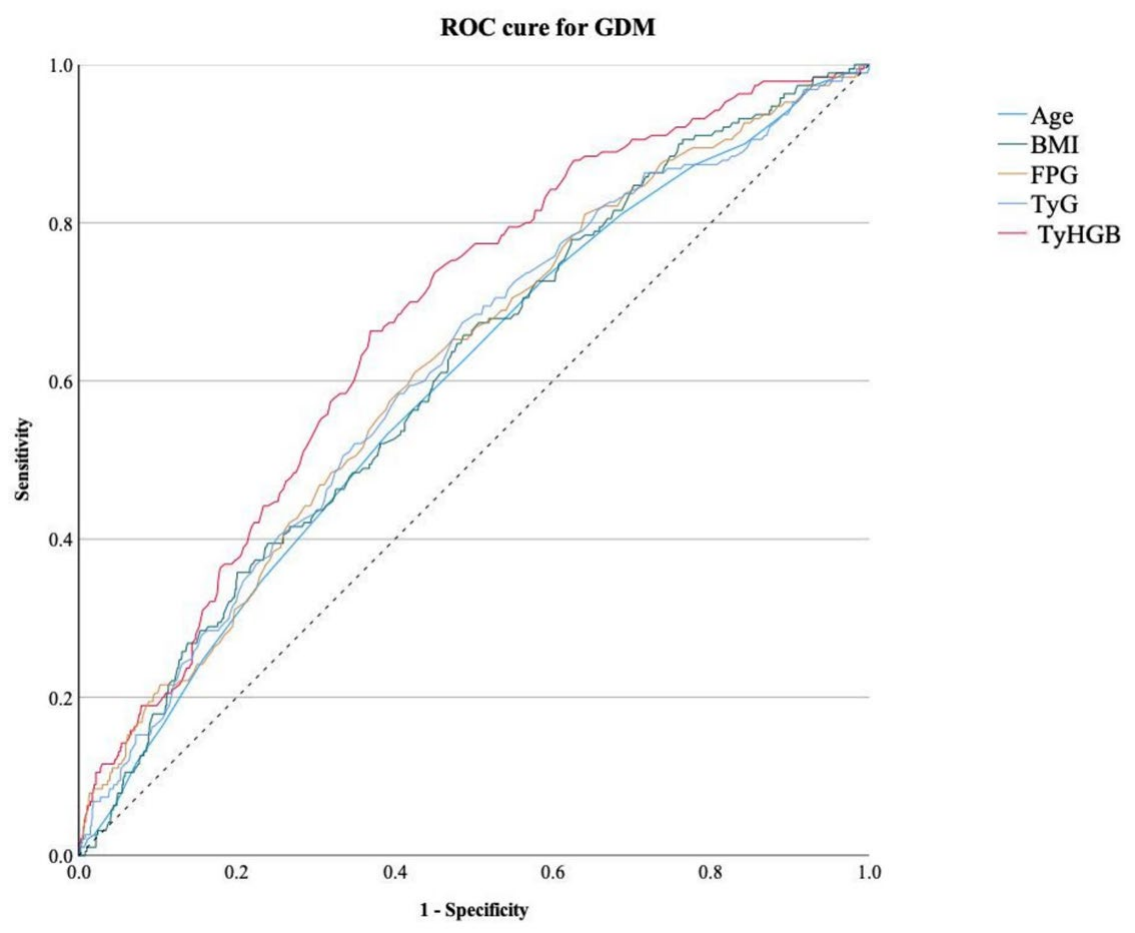
ROC curve analysis of age, BMI, FPG, TyG, and TyHGB were used to predict GDM risk.

**Table 5:** Areas under the receiver operating characteristic curves (AUROC) for each evaluated parameter in identifying GDM.

Variables	AUC	Sensitivity	Specificity	PPV	NPV	Best threshold	Youden’s index
TyHGB	0.675 (0.646, 0.703)	0.6632	0.6312	0.90 (0.87, 0.92)	0.28 (0.24, 0.32)	5.995	0.294
Age	0.596 (0.566, 0.626)	0.5316	0.6105	0.86 (0.83, 0.88)	0.23 (0.19, 0.27)	31.5	0.1421
BMI	0.610 (0.580, 0.639)	0.6579	0.5132	0.87 (0.84, 0.90)	0.23 (0.19, 0.26)	20.955	0.1711
FPG	0.618 (0.588, 0.647)	0.6105	0.5750	0.87 (0.84, 0.90)	0.24 (0.20, 0.28)	81.27	0.1856
TyG	0.615(0.585, 0.644)	0.6737	0.5143	0.88 (0.85, 0.91)	0.23 (0.20, 0.27)	8.275	0.1880

## Discussion

This retrospective cohort study investigated the association between first-trimester TyHGB index and subsequent GDM risk among pregnant women in Southern China. Our results demonstrated that higher TyHGB independently increased GDM susceptibility, exhibiting a significant linear dose–response relationship across TyHGB tertiles or quintiles. With an optimal Youden index of 5.995, TyHGB yielded an AUC of 0.675 (95% CI: 0.646–0.703), corresponding to a sensitivity of 66.32% and a specificity of 63.12%. Notably, TyHGB showed superior predictive efficacy compared with conventional risk markers, including maternal age, pre-BMI, FPG, and TyG index. These findings suggest that TyHGB may serve as a simple and clinically feasible early predictive biomarker for GDM.

Consistent with previously validated evidence, our study confirmed the independent predictive value of first-trimester TyHGB for GDM. The TyHGB index, originally proposed by Song et al., is an integrated metabolic metric derived from the TyG index, with additional incorporation of HDL-C and pre-BMI, two critical parameters closely tied to glucose homeostasis and metabolic dysfunction (24). Consistent with previous evidence, the association of TyHGB with insulin resistance in early pregnancy, with a good ability to predict GDM, was confirmed by the study of Song et al. (24). In line with our findings, prior external validation studies in Chinese and Korean pregnant populations have reported significant associations between elevated TyHGB and increased GDM risk, with OR values ranging from 1.88 to 2.09 (26). Our cohort obtained a slightly lower independent OR of 1.62 (95% CI: 1.29–2.03) after comprehensive confounding adjustment, which may be attributed to differences in geographical population backgrounds, sample size, covariate correction strategies, and gestational sampling windows across studies.

The underlying mechanism linking TyHGB to GDM is closely related to insulin resistance, which is recognized as the core pathological basis of GDM development (22). Each component of TyHGB plays an indispensable role in maintaining gestational metabolic homeostasis. The TyG index primarily reflects glycemic and triglyceride metabolic status, whereas BMI mirrors systemic adiposity, and HDL-C participates in anti-inflammatory and lipid regulatory processes. Accumulating evidence has validated the predictive value of pre-BMI (27, 28), HDL-C (29, 30), and TyG (31) for GDM. Impaired HDL-C function aggravates lipotoxicity and insulin resistance, further disrupting glucose homeostasis and facilitating GDM progression (32). A large-scale meta-analysis including over 960,000 pregnant women confirmed that increased pre-pregnancy BMI significantly elevates GDM risk, with each unit BMI increment associated with a 4% higher GDM incidence (33). In addition, the TyG index has been consistently associated with increased GDM risk across different ethnic populations, including Iranian (34), Hungarian (35), Korean (36), Mexican (37), and Chinese women (38), demonstrating its stable metabolic predictive value across ethnicities. Existing studies have also confirmed that combining traditional clinical indicators can improve GDM predictive efficiency. In line with this notion, Hantoushzadeh et al. (39) developed a prediction model using maternal age, pre-pregnancy BMI, and fasting plasma glucose, which enables convenient risk stratification for routine prenatal care. Compared with single biomarkers, TyHGB strengthens the predictive power for metabolic disorders without additional clinical costs or increased testing complexity. Collectively, the composite TyHGB index compensates for the limited accuracy of single-parameter screening models. It enables improved early identification of high-risk pregnancies, facilitates individualized risk stratification, and supports targeted early clinical monitoring and intervention.

Our subgroup analyses further highlight the clinical utility of TyHGB, particularly in low-risk pregnant populations. We observed significant interactions between TyHGB and pre-pregnancy BMI as well as gravidity. The predictive association between TyHGB and GDM remained robust among women aged <35 years, those with pre-pregnancy BMI < 25, and women with spontaneous conception, while this association was attenuated in women with pre-pregnancy BMI ≥ 25 (40). Conventional risk factors such as obesity and advanced maternal age are strong predictors in high-risk cohorts and may dominate risk assessment, thereby masking the incremental predictive value of novel glycemic biomarkers (41). In low-risk pregnant individuals without these classic risk factors, subtle early glycemic dysregulation is often difficult to capture using traditional risk assessment tools (42). In this context, TyHGB may serve as a valuable complementary predictive index for GDM screening in low-risk pregnant women, helping to identify early metabolic abnormalities for targeted surveillance (43). Although TyHGB was significantly associated with GDM risk in both nulliparous and multiparous women, the stronger effect magnitude observed in nulliparous women further underscores its potential for risk stratification in low-risk gravid populations.

Compared with existing studies focusing on first-trimester metabolic predictors of GDM, the present study has both consistent findings and unique strengths in terms of study design, population characteristics, exposure measurement, statistical analysis, and clinical interpretation. Most previous relevant studies were either single-center cohorts with limited sample size or lacked standardized first-trimester biomarker detection windows (44). In terms of exposure indicators, prior studies mainly focused on individual metabolic markers such as TyG, FPG, or BMI, while few comprehensively integrated multiple insulin resistance-related metabolic parameters to construct a composite predictive index (31, 45). In terms of statistical strategies, most previous studies only reported simple correlation or regression results without dose–response trend analysis or stratified interaction analyses (44, 46). In comparison, our study enrolled a relatively large sample of Southern Chinese pregnant women with unified first-trimester blood sampling, strictly adjusted for multiple potential confounding factors, and verified a stable linear dose–response relationship between TyHGB and GDM risk. More importantly, we further explored subgroup differences and clarified the superior predictive value of TyHGB in low-risk pregnant populations, which has rarely been discussed in previous studies. Therefore, our findings supplement and extend current evidence regarding early GDM prediction in Chinese pregnant women.

Therefore, TyHGB represents a promising, cost-effective, and widely applicable screening tool for early GDM prediction in clinical obstetric practice. Nevertheless, these subgroup findings remain exploratory. As this was a single-center retrospective cohort study, external validation in larger prospective cohorts is required before broad clinical implementation.

### Study strengths and limitations

This study adopted a rigorous retrospective cohort design, which strengthened the credibility of our findings and minimized recall bias. We comprehensively adjusted for a wide range of metabolic and clinical confounders to systematically explore the association between early-pregnancy TyHGB and GDM risk. Furthermore, subgroup analyses and stratified verification confirmed the stability and robustness of the results. Notably, TyHGB exhibited better predictive performance than conventional markers, including maternal age, pre-BMI, FPG, and TyG index, with a reliable optimal cut-off value identified in our cohort.

Nevertheless, several limitations should be acknowledged. First, this study lacked information on certain lifestyle and socioeconomic factors, such as dietary patterns, physical activity, sleep quality, psychological status, and household income, which may potentially affect glucose metabolism and GDM development. Second, TyHGB was only measured during early pregnancy, and dynamic changes throughout gestation were not monitored. Single-point measurement may fail to capture gestational metabolic fluctuations. Third, the present study exclusively enrolled singleton pregnancies; therefore, whether TyHGB remains valid for twin or multiple pregnancies requires further verification. Fourth, data on medications interfering with glucose metabolism were not systematically recorded in the retrospective medical records, which may introduce unmeasured confounding effects on the study results. Finally, although participants with major chronic diseases were excluded based on medical records, unrecorded subclinical conditions that influence glucose and lipid metabolism could not be completely ruled out, which may introduce residual confounding. In addition, the AUC of TyHGB was 0.675, indicating moderate predictive accuracy. Therefore, TyHGB cannot serve as a standalone diagnostic indicator for GDM but is suitable for auxiliary early risk stratification in clinical practice.

## Conclusion

In summary, the present study identified a positive association between parameters measured in the first-trimester (<14 gestational weeks) and subsequent GDM diagnosed at 24–28 weeks of gestation among pregnant women in Southern China. As a novel, readily-available metabolic indicator, TyHGB shows promising early-predictive performance for GDM and could be incorporated into routine antenatal screening protocols. Integrating TyHGB into early-pregnancy clinical assessment allows clinicians to recognize women at high GDM risk at an early gestational stage, supporting timely risk stratification, targeted surveillance, and proactive intervention. Such early-screening strategies may help lower GDM incidence and ultimately optimize maternal and neonatal pregnancy outcomes.

## Data Availability

The raw data supporting the conclusions of this article will be made available by the authors, without undue reservation.

## Glossary

ALT: Alanine aminotransferase
AST: Aspartate aminotransferase
AUC: Area under the receiver operating characteristic curve
BMI: Body mass index
CI: Confidence interval
FPG: Fasting plasma glucose
GDM: Gestational diabetes mellitus
HbA1c: Glycated hemoglobin
HDL-C: High-density lipoprotein cholesterol
IADPSG: International Association of the Diabetes in Pregnancy Study Groups
IQR: Interquartile range
LDL-C: Low-density lipoprotein cholesterol
OGTT: Oral glucose tolerance test
OR: Odds ratio
Pre-BMI: Pre-pregnancy body mass index
ROC: Receiver operating characteristic
TC: Total cholesterol
TG: Triglyceride
TyG: Triglyceride-glucose index
TyHGB: Triglyceride-high-density lipoprotein cholesterol-glucose body index
